# Hydropneumothorax and Pulmonary Embolism in a Patient With Shortness of Breath

**DOI:** 10.7759/cureus.27986

**Published:** 2022-08-14

**Authors:** Obinna Enemoh, Obichukwu Iwunna

**Affiliations:** 1 Emergency Medicine, Epsom and St Helier University Hospitals NHS Trust, Surrey, GBR; 2 Emergency Medicine, Mid Cheshire Hospitals NHS Foundation Trust, Crewe, GBR

**Keywords:** pneumonia, thoracentesis, pulmonary embolism, hydropneumothorax, pleural effusion, pneumothorax

## Abstract

This is a case of hydropneumothorax in an elderly man presenting to the emergency department with worsening respiratory symptoms one month after treatment for a chest infection and pleural effusion. Computerized tomography pulmonary angiogram (CTPA) showed an encysted hydropneumothorax and chronic pulmonary emboli. He was anticoagulated, had a chest drain with a good outcome and was discharged for a multidisciplinary team follow-up.

This report highlights hydropneumothorax occurring as a result of therapeutic or diagnostic pleural interventions. It also reiterates the importance of identifying the probable cause of a pleural effusion, with pulmonary embolism as a cause that should be ruled out.

## Introduction

Hydropneumothorax is the collection of both fluid and air within the pleural space. It is a rare condition, however, it has been associated with a chest infection, malignancy, chest trauma and pleural procedures like thoracentesis [1]. Pulmonary embolism is an occlusion along the pulmonary artery by an embolus. Risk factors include deep vein thrombosis, previous venous thromboembolism (VTE), active cancer, significant immobility, and advanced age. [2] If untreated, the mortality rate is high and so early identification of pulmonary embolism and commencement of treatment is vital to increasing the chances of a good outcome and preventing mortality [3].

In this case, we highlight the differentials of patients presenting with respiratory symptoms, diagnostics difficulties and complications that can arise from pleural procedures. We also suggest that the hydropneumothorax in this patient may have been a result of pulmonary embolism.

## Case presentation

A 79-year-old man presented to the emergency department with sudden-onset shortness of breath. A month prior, he was managed for right-sided lobar pneumonia complicated by pleural effusion. The pleural effusion was aspirated, and the aspirate cytology analysis was negative for malignant tissue. He was discharged in stable condition and awaiting an outpatient chest CT scan until re-presentation. He is an ex-smoker with no other significant past medical history.

On re-presentation in the emergency department, he was dyspnoeic with a respiratory rate of 32 cycles per minute, hypoxic with oxygen saturation of 84% on room air and tachycardic with a heart rate of 106 beats per minute. He had unequal chest wall movement; absent breath sounds in the middle and lower zones of the right lung and dull percussion on the same side.

**Figure 1:**
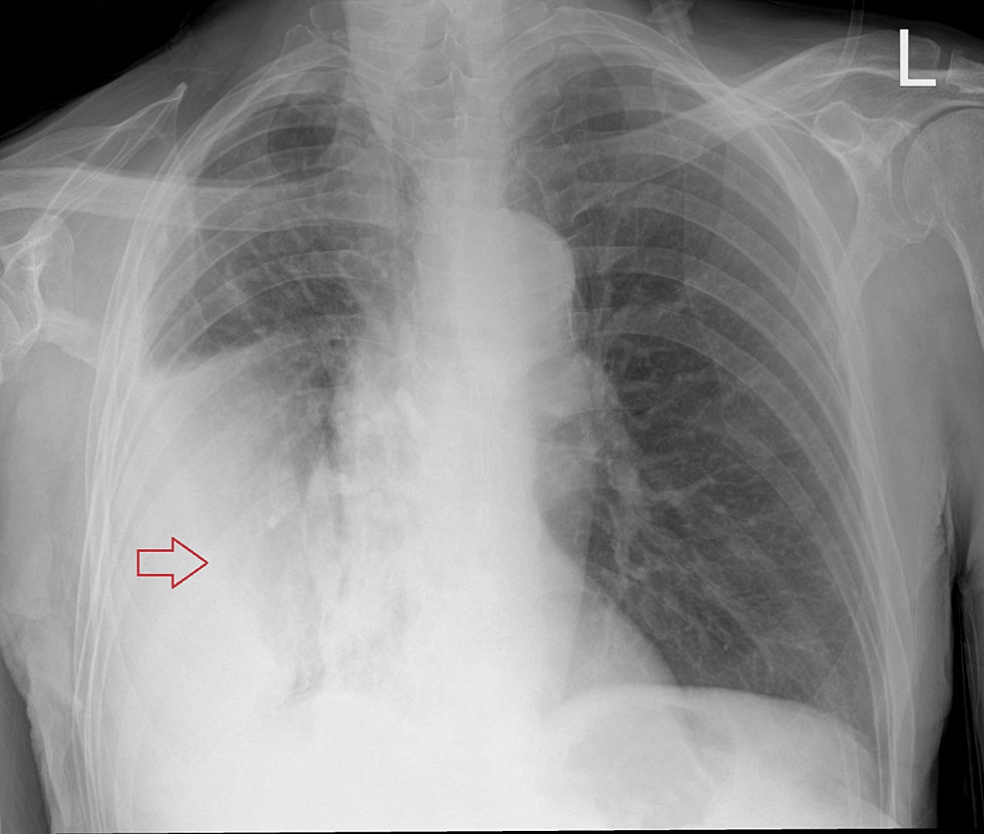
Chest radiograph shows pleural effusions with air-fluid levels in the right hemithorax (red arrow)

Laboratory results showed markedly increased white cell count of 17.9 x 10*9/L and raised C-reactive protein (68.6 mg/L). This was in line with active pneumonia for which he was taking oral antibiotics. D-dimer levels, however, were dramatically high (>6000 ng/mlFEU). A 12-lead ECG on this patient showed sinus tachycardia and his Well’s score was 4.5. A chest radiograph (Figure 1) done showed effusions on the right hemithorax with air leading to a diagnosis of a right-side hydropneumothorax.

He was commenced on oxygen via a 28% venturi mask (with target saturations 88-92%) and intravenous fluids. In view of a diagnosis of pneumonia, he was treated with intravenous benzylpenicillin and clarithromycin according to the hospital’s guidelines. Given his clinical presentation, his Well’s score of 4.5 and a D-Dimer of >6,000, he had interim anticoagulation with low molecular weight heparin (LMWH) dalteparin while awaiting a CTPA to make a definitive diagnosis.

**Figure 2:**
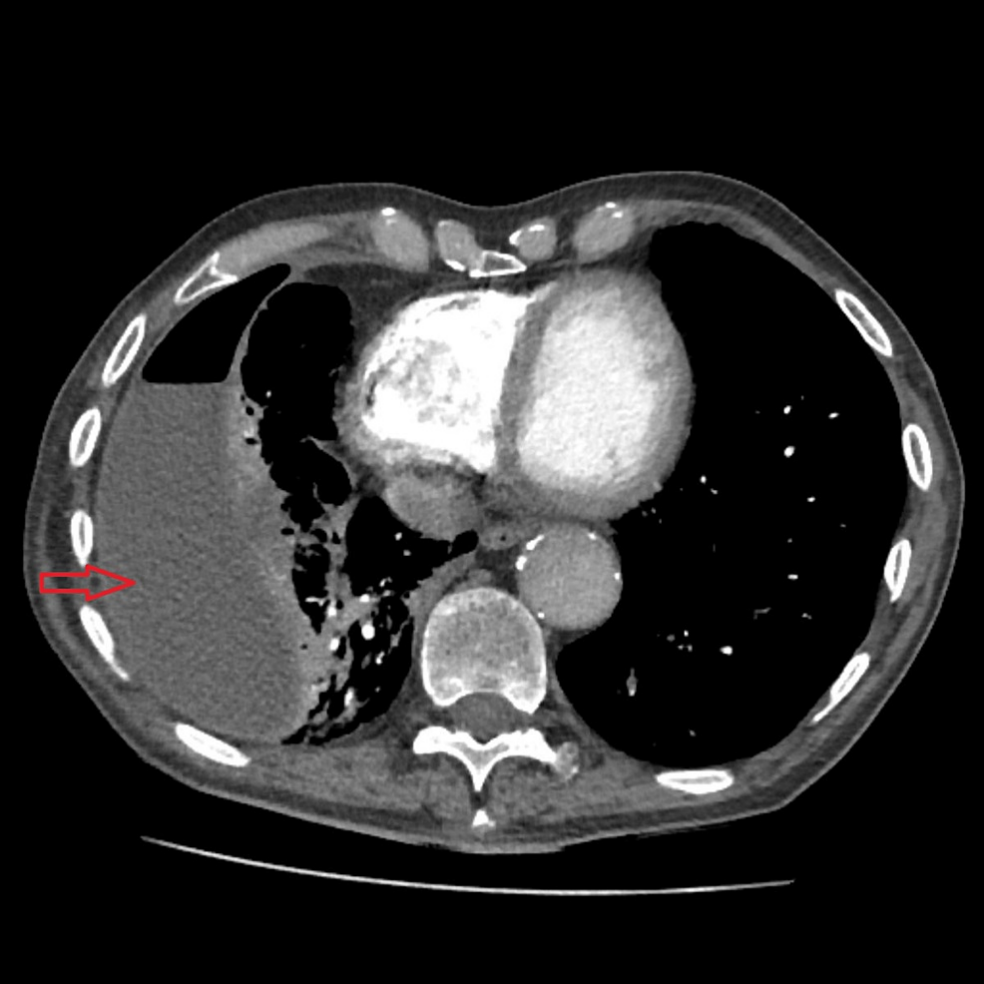
CTPA show right-sided encysted hydropneumothorax, consolidation within the right middle and lower lung zones (red arrow) and small bilateral pulmonary emboli CTPA: computerized tomography pulmonary angiogram

**Figure 3:**
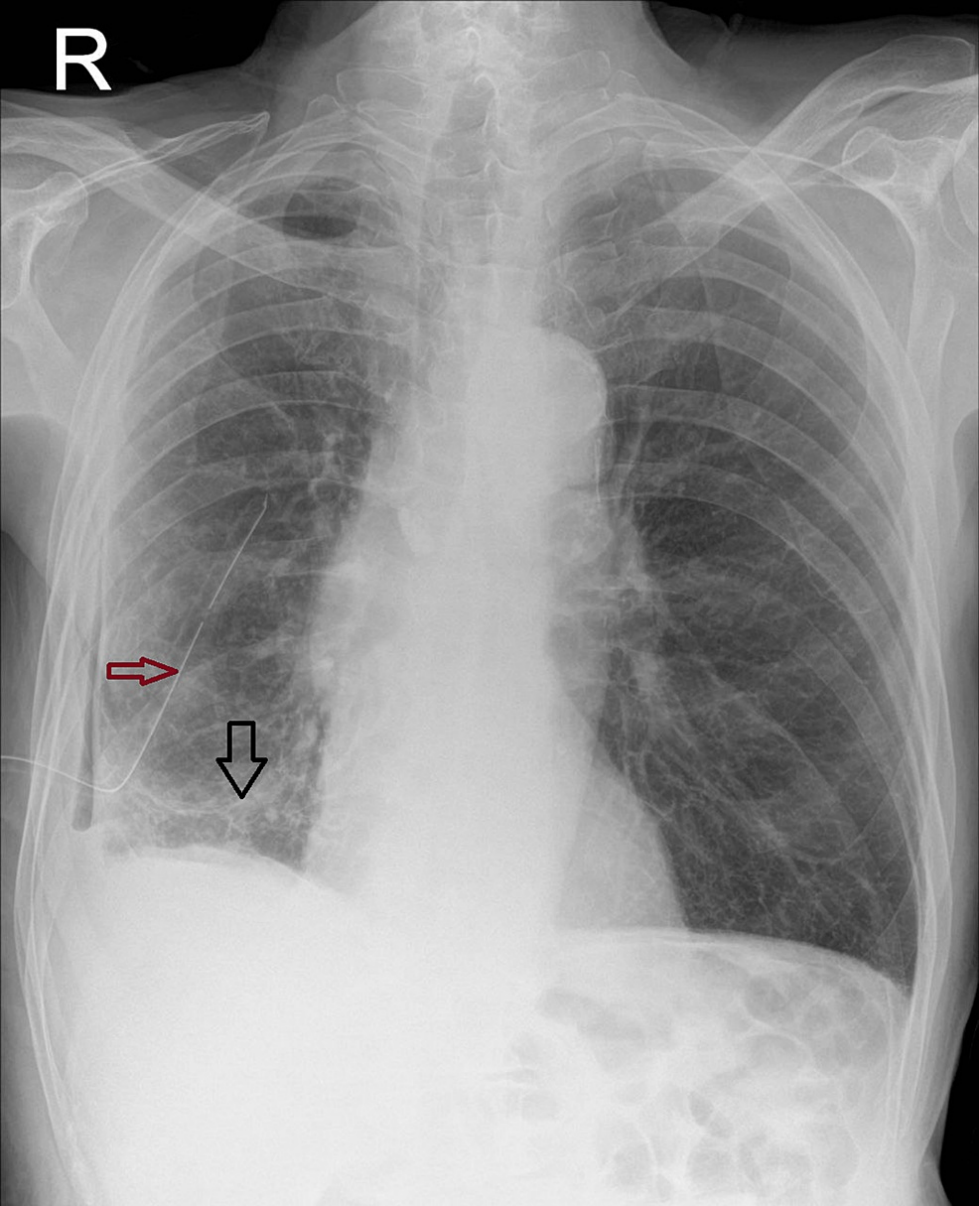
Chest radiograph with a chest tube in situ (red arrow) showing lung re-expansion (black arrow)

Further assessment with a CTPA (Figure 2) revealed bilateral small chronic pulmonary emboli, right-sided encysted hydropneumothorax, atelectasis and consolidation within the middle and lower zones of the right lung with broncho-centric thickening. The patient was managed with treatment doses of dalteparin for the pulmonary embolism, admitted and had a chest drain inserted while on admission with good outcomes. Repeat chest radiographs showed steady lung re-expansion (Figure 3), and he was discharged to have a multidisciplinary team (MDT) follow-up.

## Discussion

Hydropneumothorax is a rare condition with varying aetiologies [4]. This case highlights some of these causes that include a complication of inflammatory processes and iatrogenic causes like thoracocentesis. Our patient had pneumonia, which was complicated by pleural effusion. He also had thoracocenteses and pleural fluid analysis. His recovery was cut short by worsening shortness of breath and on this occasion, his chest radiograph showed both fluid and air levels. While inflammatory changes can lead to structural alterations in the lung parenchyma, which ultimately lead to the development of a pneumothorax, pleural procedures, such as thoracocentesis, in this case, are an established cause [1]. We postulate that the hydropneumothorax, in this case, was a complication of the thoracentesis done.

Pulmonary embolism is acknowledged as a leading cause of pleural effusion and is highly suggestive of pleuritic chest pain accompanied by pleural effusion [5]. CTPA done in the current presentation showed chronic pulmonary emboli. While the initial managing clinicians thought the patient’s pleural effusion was a complication of his pneumonia, we believe a pulmonary embolus might be a significant causative factor. It is possible that the emboli were missed at his presentation the month before, as it was not ruled out in that presentation due to the hospital’s protocols. It is, therefore, vital to have a high index of suspicion for a pulmonary embolism in a patient presenting with pleural effusion and possibly rule that out prior to discharge.

An important differential for shortness of breath is malignant processes. The pathological processes (pleural effusion, pneumothorax and pulmonary embolism) in our patient can be explained by a malignancy; however, pleural aspirate cytology was negative for malignant cells. There is still a strong suspicion of malignancy regardless, and he will have a multidisciplinary team meeting on discharge.

It is therefore important to identify the cause, as that will determine definitive management and improve outcome.

## Conclusions

This case report draws attention to hydropneumothorax occurring as a complication of therapeutic interventions and inflammatory processes. More importantly, it highlights how vital it is for clinicians to identify the probable cause of pleural effusion and provide definitive management based on the identified cause. Pulmonary embolism is an important differential diagnosis for shortness of breath, which should be excluded in the presence of risk factors, as it may be a cause of the complications.
